# Bioactive derivatives of the antimicrobial peptide esculentin-1a promote human dermal fibroblast migration and activate genes involved in early wound healing

**DOI:** 10.1016/j.bbadva.2026.100186

**Published:** 2026-03-28

**Authors:** Floriana Cappiello, Eleonora Grisard, Alice Traversa, Danilo Ranieri, Maria Luisa Mangoni

**Affiliations:** a Laboratory Affiliated to Pasteur Italia-Fondazione Cenci Bolognetti, Department of Biochemical Sciences, Sapienza University of Rome, 00185 Rome, Italy; b Department of Life Science, Health, and Health Professions, Link Campus University, 00165 Rome, Italy

**Keywords:** Antimicrobial peptides, Human dermal fibroblasts, Wound healing, MAPK/ERK pathway

## Abstract

Antimicrobial peptides (AMPs) are essential components of the innate immune system and have emerged as promising candidates against antibiotic-resistant infections. Beyond their antimicrobial activity, AMPs also play a crucial role in regulating tissue regeneration processes, including wound healing. Previous studies have shown that the frog skin-derived AMP, esculentin-1a(1-21) [Esc(1-21)], promotes wound healing by accelerating keratinocyte migration. Its derivative, esculentin-1a(1-21)-1c [Esc(1-21)-1c] has proven even more effective in facilitating wound closure in alveolar and bronchial epithelial cell monolayers. Given the essential role of dermal fibroblasts in skin regeneration, this study aimed to investigate the effects of Esc(1-21) and its two derivatives, Esc(1-21)-1c and [Aib^8^]-Esc(1-21) (containing the non-natural α-aminoisobutyric acid at position 8), on the activation of human primary dermal fibroblasts (hDFs) during the healing of a pseudo-wound. The results demonstrated that all three peptides significantly enhanced hDFs migration and stimulated wound closure by activating the MAPK/ERK signaling pathway, without affecting cell proliferation. Importantly, for Esc(1-21) and [Aib^8^]-Esc(1-21), this activation was clearly mediated by Epidermal Growth Factor Receptor (EGFR). Finally, the peptides upregulated the expression of early wound healing markers, such as Collagen I and Collagen III, but did not alter the expression of late-stage markers, including MMP9 and α-SMA. These findings highlight the regenerative potential of Esc(1-21) and its derivatives and suggest their relevance in therapeutic strategies for impaired wound healing.

## Highlights

• Esculentin-1a derivatives stimulate fibroblast migration during wound healing.
• Peptides-induced fibroblast migration involves the activation of MAPK/ERK pathway.
• Esculentin-1a derivatives upregulate early wound healing gene expression.

## Graphical abstract

**Figure:**
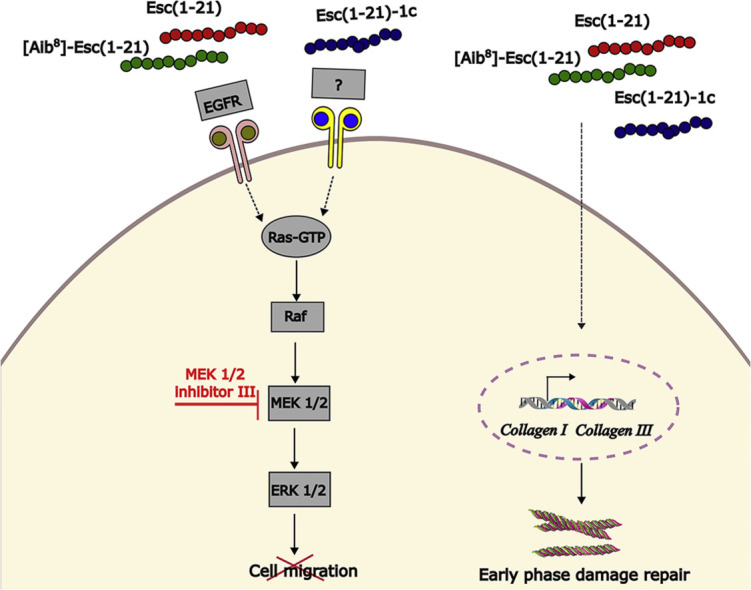

## 1 Introduction

The skin is one of the largest organs in the human body consisting of epidermis, dermis, and subcutaneous tissue or hypodermis [1]. As the outermost layer of the skin, the epidermis serves as a semi-permeable barrier, acting as the body's first line of defense against external factors encompassing invasive pathogenic microorganisms, chemical and physical agents [2,3]. It also helps prevent dehydration [4]. In comparison, the intermediate layer, the dermis, contributes to skin support and flexibility, and it is rich in blood vessels, nerve fibers, lymphatic systems, collagen fibers and fibroblasts [5,6]. Following accidental trauma, surgery, microbial infections, burns, or conditions such as diabetes, the integrity of the skin can be compromised, leading to the formation of wounds [[2], [3], [4], [5], [6]]. The process by which the body restores the integrity and physiological functions of damaged tissue in response to a destructive stimulus is known as wound healing [2,[7], [8], [9]]. During this complex and vital biological process, various intracellular and intercellular pathways must be activated and precisely coordinated. Additionally, multiple cell types are recruited, including fibroblasts, which play a crucial role in the deposition and remodeling of extracellular matrix (ECM) components, such as collagen [5,10,11]. Unfortunately, delay or failure in the mechanisms responsible for tissue restoration can induce the formation of chronic or non-healing wounds, significantly impacting the healthcare system [[12], [13], [14]]. This is particularly concerning in cases where bacterial biofilms form, exhibiting resistance to conventional antibiotics [[15], [16], [17], [18]]. The Gram-negative bacterium *Pseudomonas aeruginosa* and the Gram-positive bacterium *Staphylococcus aureus* are the most prevalent microbial strains responsible for the formation of biofilms in chronic or non-healing wounds [[17], [18], [19]]. Therefore, the development of alternative treatment strategies for infected wounds, capable of both combating infections and accelerating tissue healing, is highly pressing [19].

In this regard, antimicrobial peptides (AMPs) have been proposed as promising new weapons [20]. AMPs are naturally produced by most living organisms as key components of the innate immune system [21]. Amphibian skin is one of the major sources of bioactive peptides with diverse pharmacological properties, encompassing antimicrobial, immunomodulatory and wound healing activities [16,22]. Our previous studies focused on the characterization of the frog skin-derived AMP esculentin-1a(1-21) [Esc(1-21)] [GIFSKLAGKKIKNLLISGLKG-NH_2_] [23,24]. This peptide adopts an α-helical structure in membrane mimicking environments and has a rapid bactericidal activity against both the planktonic and sessile forms of *P. aeruginosa* [25]. However, it is not effective against *S. aureus* [26,27]. Furthermore, it was found to significantly support human keratinocyte migration, enhancing wound re-epithelialization more effectively than human AMP LL-37 [28]. Over the past few years, a diastereomer of Esc(1-21), namely Esc(1-21)-1c, was designed by substituting two amino acids (L-Leu14 and L-Ser17) with their corresponding D-enantiomers. This modification aimed to reduce the α-helical content of the parental peptide, thereby decreasing its cytotoxic effects towards mammalian cells, while enhancing its resistance to proteolytic degradation [23,29]. Interestingly, Esc(1-21)-1c was able to accelerate the closure of a wounded area, produced in a monolayer of alveolar or bronchial epithelial cells, at concentrations lower than those needed for Esc(1-21) [29,30]. Subsequently, an analogue of Esc(1-21) containing the non-natural helicogenic α-aminoisobutyric acid (Aib) at position 8, i.e. [Aib^8^]-Esc(1-21), revealed to be active also against the planktonic and sessile forms of *S. aureus*, with an improved biostability than the native Esc(1-21) without displaying cytotoxicity [31].

In this work, the capability of Esc(1-21) and Esc(1-21)-1c (reported to as Esc peptides), and [Aib^8^]-Esc(1-21) to foster healing of wounds extended into deeper layers of the skin, was investigated. To this aim, the effect of these peptides was studied for the first time on the migration of human dermal fibroblasts (hDFs) in an *in vitro* cell culture model. In addition, the activation of the MAPK/ERK pathway, that is reported to exhibit a central role in several cellular processes [10,11,32], was examined to gain insight into the molecular mechanisms regulating migration of hDFs stimulated by these peptides.

## 2 Materials and methods

### 2.1 Materials

All reagents and antibodies used in this study are listed in Table 1 together with complete supplier information (supplier, city, country).

**Table 1:** List of reagents used for experimental procedures with complete reagent name and detailed supplier information.

Reagent	Supplier	Town	Country
4-(2-hydroxyethyl)-1-piperazineethanesulfonic acid (HEPES)	Sigma-Aldrich	St Louis	USA
4′,6-diamidino-2-phenylindole dihydrochloride (DAPI)	Sigma-Aldrich, Merck KGaA	Darmstadt	Germany
Anti-EGFR	Bioss	Woburn	USA
Anti-HSP90	Proteintech	Rosemont	USA
Anti-Ki67	Zymed Laboratories	South San Francisco	USA
Anti-MMP9	Santa Cruz	Dallas	USA
Anti-p44/42 (ERK 1/2)	Cell Signaling	Danvers	USA
Anti-phospho EGFR	Cell Signaling	Danvers	USA
Anti-phospho p44/42 (ERK 1/2)	Cell Signaling	Danvers	USA
Anti-phospho STAT3	Cell Signaling	Danvers	USA
Anti-STAT3	Cell Signaling	Danvers	USA
Anti-α-SMA	Sigma-Alrich	St Louis	USA
Anti-β-ACTIN	Santa Cruz	Dallas	USA
Cell counting Kit-8 (CCK8)	MedChem Express	New Jersey	USA
cOmplete™ protease inhibitor cocktail tabs	Roche	Basel	Switzerland
Dulbecco’s modified Eagle Medium (DMEM)	Sial	Rome	Italy
Ethylene glycol tetraacetic acid (EGTA)	Sigma-Aldrich	St Louis	USA
Fetal Bovine Serum (FBS)	Sial	Rome	Italy
Fluorescein Isothiocyanate (FITC)-phalloidin	Sigma-Aldrich, Merck KGaA	Darmstadt	Germany
Glycerol	Sigma-Aldrich	St Louis	USA
Ibidi culture inserts	Ibidi	Gräfelfing	Germany
iScript TM cDNA syntesis Kit	Bio-Rad	Hercules	USA
MEK 1/2 inhibitor III (PD0325901)	Calbiochem	San Diego	USA
MgCl_2_	Thermo Scientific Chemicals	Waltham	USA
Mitomycin C	Sigma-Aldrich	St Louis	USA
Mowiol	Sigma-Aldrich	St Louis	USA
NaCl	Sigma-Aldrich	St Louis	USA
Nitrocellulose	Schleider&Schuell BioScience	Keene	USA
Penicillin, Streptomycin	Sial	Rome	Italy
PhosSTOP™	Roche	Basel	Switzerland
Phosphate-buffered saline (PBS)	Sial	Rome	Italy
Quick RNA™Microprep Kit	Zymo Research	Irvine	USA
SYBR green	Bio-Rad	Hercules	USA
Triton X-100	Sigma-Aldrich	St Louis	USA

### 2.2 Peptides

Esc(1-21), Esc(1-21)-1c and [Aib^8^]-Esc(1-21) analogue were purchased from Biomatik (Wilmington, USA). Their primary structure is reported in Table 2. Peptides were synthesized by stepwise solid-phase peptide synthesis using the Fmoc strategy and purified by reverse-phase high-performance liquid chromatography (RP-HPLC) using a gradient of acetonitrile, with UV detection at 220 nm, to give purities of 96–97 %. The molecular mass was confirmed using mass spectrometry.

**Table 2:** Primary structure of Esc(1-21) and its analogues.

PEPTIDE DESIGNATION	SEQUENCE^a^	Observed MW (g/mol)	Expected MW (g/mol)
Esc(1-21)	GIFSKLAGKKIKNLLISGLKG-NH₂	2185.40	2184.71
Esc(1-21)-1c	GIFSKLAGKKIKN*L*LI*S*GLKG-NH₂	2185.20	2184.71
[Aib^8^]-Esc(1-21)	GIFSKLAAibKKIKNLLISGLKG-NH_2_	2212.80	2212.76

a Residue variations compared to Esc(1-21) are in bold. D-amino acids are underlined. Aib, aminoisobutyrric acid. Expected MW was calculated using PepCalc.com (https://pepcalc.com/)

### 2.3 Cell culture

Primary cultures of human fibroblasts derived from healthy skin (hDFs) were obtained from patients attending the Dermatology Unit of the Sant’Andrea Hospital of Rome; all patients were extensively informed and their consent for the investigation was given and collected in written form in accordance with guidelines approved by the management of the Sant’Andrea Hospital. Primary cells were isolated and cultured as previously described [33]. Cells were cultured at 37°C and 5 % CO_2_ in Dulbecco’s modified Eagle Medium (DMEM) supplemented with 2 mM glutamine, 10 % fetal bovine serum (FBS) and antibiotics (0.1 mg/mL of streptomycin and penicillin).

### 2.4 Cell viability assay

To evaluate hDFs viability, the Cell Counting Kit-8 (CCK-8) assay (MedChemExpress, New Jersey, USA) was performed. Briefly, about 1 × 10^4^ cells, in 100 μL of DMEM supplemented with 10 % FBS, were added to each well of a 96-well plate and incubated at 37°C and 5 % CO_2_. After approximately 24 hours the medium was discarded and replaced with 100 μL of DMEM supplemented with each peptide at different concentrations (ranging from 4 μM to 128 μM). The plate was incubated for 24 or 48 hours at 37°C and 5 % CO_2_. Then, according to manufacturer's protocol, 10 μL of CCK-8 solution was added to each well and after 2 hours incubation the absorbance of each well was measured at 450 nm using a microplate reader (Infinite M200; Tecan, Salzburg, Austria). The number of living cells is directly proportional to the quantity of orange formazan generated by intracellular dehydrogenase activity. The cell viability was expressed as a percentage with respect to the untreated control cells.

### 2.5 Pseudo-wound healing assay

To assess the peptides’ ability to stimulate cell migration, a modified scratch assay was performed [34]. An Ibidi culture insert (Ibidi, Gräfelfing, Germany) was placed in each well of a 24-well plate and 2.5 × 10^4^ cells, suspended in DMEM plus 10 % FBS, were seeded in each compartment of the inserts. Subsequently, the plate was incubated at 37°C and 5 % CO_2_ for approximately 24 hours to allow cells to reach confluence and when the insert was removed, a cell-free area (pseudo-wound) of approximately 500 μm between two cell monolayers was produced. Different concentrations of each peptide in DMEM supplemented with 1 % FBS were added to the cells and plates were incubated as described above to allow cells to migrate. Samples were visualized at different time intervals under an inverted microscope (Olympus CKX41, Olympus, Tokyo, Japan) at 4x magnification and photographed with a Color View II digital camera. The percentage of scratch closure at each time was determined by the program FastTrack Al™ Metavì Labs.

Pseudo-wound healing assays were also performed by pre-treating hDFs cells with 30 μM mitomycin C (Sigma-Aldrich, St Louis, USA) for 2 hours [11] or with 1 μM MEK1/2 inhibitor III, PD0325901 (Calbiochem, San Diego, USA) for 1 hour [10], in order to assess the contribution of cell proliferation or the involvement of ERK phosphorylation in the peptide-induced migration of fibroblasts, respectively. After pre-treatment, MEK1/2 inhibitor III was incubated with each peptide. In parallel, samples treated with single peptide or inhibitors alone were also included for comparison.

### 2.6 Fluorescence microscopy

About 2.5 × 10^4^ hDFs were seeded in each compartment of the culture insert mentioned above, placed on 0.13- to 0.17-mm-thick coverslips previously put into 35-mm dish plates. After approximately 24 hours incubation at 37°C and 5 % CO_2_, the inserts were removed to form the cell monolayers and each peptide at 10 μM concentration was added in DMEM supplemented with 1 % FBS for 24 hours. Cells maintained in the medium alone were used as control. Afterwards, cell monolayers were fixed with 4 % formaldehyde for about 10 min and permeabilized with 0.1 % Triton X-100 (Sigma-Aldrich, St Louis, USA) in phosphate-buffered saline (PBS) (Sial, Rome, Italy) for 5 min, at room temperature. Then, cells were stained with fluorescein isothiocyanate (FITC)-phalloidin (Sigma-Aldrich, Merck KGaA, Darmstadt, Germany) (40 μM in PBS) for 20 min and with 4′,6-diamidino-2-phenylindole dihydrochloride (DAPI) (Sigma-Aldrich, Merck KGaA, Darmstadt, Germany) (1 μg/mL) for 5 min at room temperature to visualize the cytoskeleton and the nuclei, respectively. Coverslips were finally mounted with Mowiol solution (Sigma-Aldrich, St Louis, USA). Fluorescence signals were analyzed with an ApoTome System (Zeiss, Oberkochen, Germany), image analysis was performed by the Axiovision software (Zeiss Oberkochen, Germany) as reported [35].

### 2.7 Cell proliferation studies

About 1.5 × 10^4^ cells in complete medium were seeded on 0.13- to 0.17-mm-thick coverslips (properly put into each well of a 24-well plate). After approximately 24 hours incubation at 37°C and 5 % CO_2_, the medium was aspirated, and some cells were pretreated with 30 μM mitomycin C for 2 hours in DMEM supplemented with 1 % FBS. Subsequently, the medium was discarded and fresh DMEM + 1 % FBS containing each peptide at 10 μM concentration was added for 48 hours and cells were incubated at 37°C and 5 % CO_2_. Afterwards, cells were washed three times with PBS and fixed with 4 % formaldehyde for 10 min at room temperature. Then, cells were washed twice with PBS and stained with an anti-Ki67 rabbit polyclonal antibody (1:50; Zymed Laboratories, South San Francisco, USA) and with DAPI (1 μg/mL) for 5 min at room temperature to visualize the nuclei. Finally, coverslips were mounted with Mowiol in PBS for observation. Fluorescence signals were analyzed by conventional fluorescence with an ApoTome System (Zeiss, Oberkochen, Germany) connected with an Axiovert 200 microscope (Zeiss, Oberkochen, Germany); image analysis was then performed by a software Axiovision (Zeiss, Oberkochen, Germany).

### 2.8 RNA extraction and cDNA synthesis

About 1 × 10^5^ hDFs cells in complete medium were seeded in 35-mm dish plates and incubated at 37°C and 5 % CO_2_. The following day, cells were serum starved for 6 hours and then treated or not with each peptide at 10 μM concentration. After 12 hours incubation at 37°C and 5 % CO_2_, total RNA was extracted using the Quick-RNA™ Microprep Kit (Zymo Research, Irvine, USA) by applying standard protocol. Total RNA concentration was quantified by the Nanodrop 1000 (Thermo Fisher Scientific, Waltham, USA); 1 µg of total RNA was reverse transcribed using the iScriptTM cDNA Synthesis Kit (Bio-Rad, Hercules, USA), with a mixture of oligo (dT) and random primers according to manufacturer’s instructions.

### 2.9 PCR amplification and real-time quantitation

qRT-PCR was performed in triplicate with SYBR Green (Bio-Rad, Hercules, USA), using 15 ng of cDNA, on a IQ5 real-time PCR detection System (Bio-Rad, Hercules, USA). The reaction was carried out in a 96-well plate adding forward and reverse primers for each gene and 7 μL of diluted template cDNA to a final reaction volume of 15 μL. All assays included negative control and were replicated three times. The thermal cycling program was performed as described previously [36]. The data were analyzed using the 2^−ΔΔCt^ method. Results are reported as mean values ± standard deviation (SD) from three different experiments in triplicate.

### 2.10 Primers

The oligonucleotide primers used to evaluate target and housekeeping genes, chosen using the online tool Primer-BLAST, were the following: for *COL1A1* target gene, 5’-ACATGTTCAGCTTTGTGGACCTCCG-3’(sense), 5’-ACGCAGGTGATTGGTGGGATGTCT-3’ (antisense); for *COL1A3* target gene, 5’-AGGGTGTCAAGGGTGAAAGTGGGA-3’(sense), 5’- GGGCGAGGACCATAGAGGT-3’ (antisense); for *MMP9* target gene, 5’-CGCGCTGGGCTTCGATCATT-3’(sense), 5’- GGGCGAGGACCATAGAGGT-3’ (antisense); for the *ACTA2* gene, 5’-GCACCCCTGAACCCCAAG-3’ (sense), 5’ACGATGCCAGTTGTGCGT-3’ (antisense), for the *18 S* rRNA housekeeping gene 5’-AACCAACCCGGTCAGCCCCT-3’(sense), 5’-TTCGAATGGGTCGTCGCCGC-3’ (antisense).

### 2.11 Western blot analysis

hDFs cells (3 × 10^5^ in complete medium) were seeded in 60-mm dish plates and incubated at 37°C and 5 % CO_2_ for approximately 24 hours. Subsequently, cells were serum-starved for 6 hours and then treated or not with each peptide at 10 μM concentration. After 10 min or 24 hours incubation at 37°C and 5 % CO_2_, cells were washed twice with PBS and lysed in a buffer containing 50 mM (4-(2-hydroxyethyl)-1-piperazineethanesulfonic acid (HEPES) (Sigma-Aldrich, St Louis, USA), pH 7.5, 150 mM NaCl, 1 % glycerol (Sigma-Aldrich, St Louis, USA), 1 % Triton X-100 (Sigma-Aldrich, St Louis, USA), 1.5 mM MgCl_2_, 5 mM ethylene glycol tetraacetic acid (EGTA) (Sigma-Aldrich, St Louis, USA), supplemented with protease inhibitors (cOmplete™ protease inhibitor cocktail tablets, Roche, Basel, Switzerland), and phosphatase inhibitors (PhosSTOP™ , Roche, Basel, Switzerland). A range between 20 and 50 µg of total protein was resolved under reducing conditions by 8 or 12 % SDS-PAGE (Invitrogen, Carlsbad, USA) and transferred to reinforced nitrocellulose (Schleider and Schuell, Keene, USA). The membranes were blocked with 5 % nonfat dry milk in PBS 0.1 % Tween 20 and incubated with anti-EGFR (Bioss, Woburn, USA), Phospho-EGFR (Cell Signaling, Danvers, USA), p44/42 MAPK (ERK1/2) (Cell Signaling, Danvers, USA), Phospho-p44/42 MAPK (ERK 1/2) (Cell Signaling, Danvers, USA), STAT3 (Cell Signaling, Danvers, USA), Phospho-STAT3 (Cell Signaling, Danvers, USA), HSP90 (Proteintech, Rosemont, USA), β-actin (Santa Cruz, Dallas, USA), α-SMA (Sigma-Aldrich, St Louis, USA), MMP9 (Santa Cruz, Dallas, USA). For experimental settings with MEK 1/2 inhibitor III, cells were first pre-treated with 1 μM MEK1/2 inhibitor III for 1 hour and then each peptide was added in combination with the inhibitor for 10 min. Successively, cell lysates were collected and treated as described in [37].

### 2.12 Statistical analysis

Quantitative data derived from independent experiments were expressed as the mean ± standard error of the mean (SEM) or standard deviation (SD). Statistical significance was determined using one- or two-way analysis of variance (ANOVA) with PRISM software (GraphPad, San Diego, USA). One-way ANOVA was used to analyze data presented in Figs. 5, S2 and S3, whereas two-way ANOVA was used for the data shown in Figs. 2, 4, 6, 7 and S1. P values of <0.05 were assumed to be statistically significant. The levels of statistical significance are indicated in the legend to figures.

## 3 Results

### 3.1 Peptides’ effect on the viability of human dermal fibroblasts

**Fig. 1:**
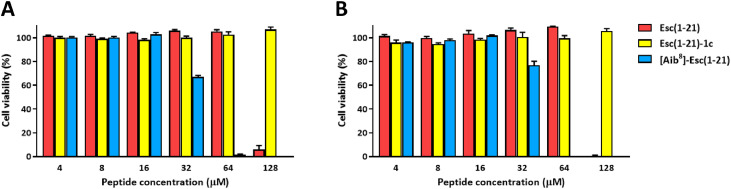
Effect of peptides on the viability of hDFs. Cell viability was monitored by the CCK-8 assay following 24 hours (A) and 48 hours (B) of treatment with different concentrations of Esc(1-21), Esc(1-21)-1c or [Aib^8^]-Esc(1-21). Cells viability is expressed as percentage compared to the control (cells not treated with the peptide, 100 % viability). All data are the means from three independent experiments ± standard error of the mean (SEM).

Esc(1-21) and its two analogues were initially assessed for their effect on the viability of hDFs by the CCK-8 assay. As shown in Fig. 1, Esc(1-21) exhibited no cytotoxic effects up to the concentration of 64 μM, after both 24 and 48 hours of treatment. However, cytotoxicity became pronounced at 128 μM, resulting in almost complete loss of viable cells. The [Aib^8^]-Esc(1-21) analogue did not show cytotoxicity between 4 μM and 16 μM; at 32 μM, cell viability was approximately 70 % and 77 %, after 24 and 48 hours, respectively, while in samples treated with 64 μM of [Aib^8^]-Esc(1-21), almost no viable cells were detected. Interestingly, the diastereomer Esc(1-21)-1c did not exhibit any cytotoxic effect at any of the tested concentrations, both after 24 and 48 hours of treatment. Dose–response analysis further indicated that [Aib^8^]-Esc(1-21) follows a typical sigmoidal profile, allowing estimation of a Lethal Concentration 50% (LC₅₀) in the range of ∼35–40 µM (data not shown). In contrast, Esc(1-21) maintained near-complete cell viability up to 64 µM, followed by a sharp drop at 128 µM, preventing a reliable LC₅₀ determination through standard nonlinear regression models.

### 3.2 Peptides’ effect on the migration of human dermal fibroblasts

Among the various cell types involved in wound healing, fibroblasts play a pivotal role by migrating to the injury site and promoting effective tissue repair [38].

**Fig. 2:**
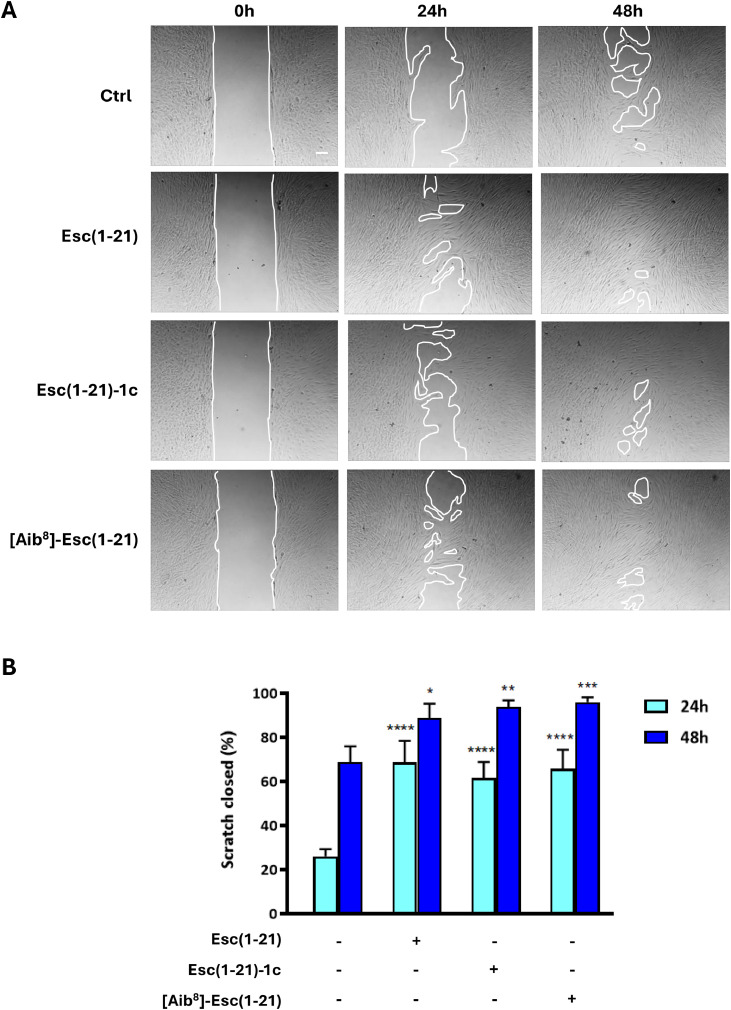
Impact of peptides’ treatment on the migration of hDFs. (A) Representative images from the modified scratch assay showing the effects of treatment of fibroblast monolayers with 10 µM Esc(1-21), Esc(1-21)-1c or [Aib^8^]-Esc(1-21). Peptide-untreated cells were used as a control (Ctrl). Scale bar: 100 µm. (B) Scratch area closure (%) in fibroblast monolayers, measured after 24 and 48 hours treatment with 10 µM peptides to evaluate cell migration. The data are the mean from four independent experiments ± SEM. The levels of statistical significance between Ctrl and treated samples are indicated as follows: p values <0.05 (*), <0.01 (**), <0.001 (***), and <0.0001 (****).

The effect of the selected peptides on fibroblast cells migration was evaluated using a modified scratch assay, in cell culture medium supplemented with 1 % FBS, at the non-toxic concentrations of 4 μM (Fig. S1) and 10 μM (Fig. 2), consistent with those previously tested on human keratinocytes [28]. The results in Fig. 2 and Fig. S1 demonstrate that all the three AMPs stimulated fibroblasts’ migration by enhancing scratch closure. The almost complete closure of the pseudo-wound field was observed for all peptides after 48 hours of treatment, at the optimal concentration of 10 μM compared to the untreated control samples (Fig. 2).

**Fig. 3:**
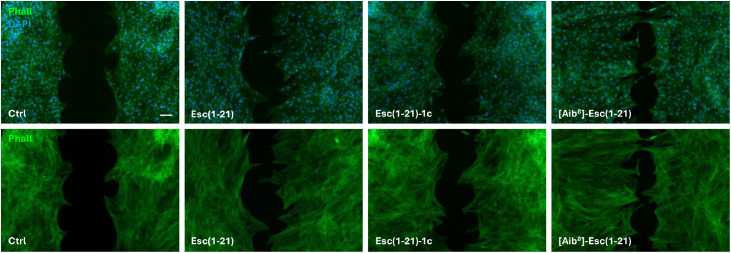
Effect of peptides’ treatment on hDFs morphology during wound healing. Micrographs showing the wound edge of hDFs after 24 hours treatment with 10 µM Esc(1-21), Esc(1-21)-1c or [Aib^8^]-Esc(1-21) compared to Ctrl. Cells were stained with DAPI to visualize cell nuclei and with FITC-phalloidin to visualize cytoskeleton. Scale bars: 30 μm. Upper panels, combination of DAPI and FITC-phalloidin staining, time 24 hours; lower panels, FITC-phalloidin staining, time 24 hours.

These results were further confirmed by visualizing the edge of the cell monolayer facing the pseudo-wound area, corresponding to the cell migration front. Samples were observed prior to the complete closure of the scratched area. As shown in Fig. 3, stimulation with the three peptides for 24 hours at the concentration of 10 μM induced a strong migratory phenotype in cells, at the scratch edge, characterized by an elongated shape, with lamellipodia and membrane ruffles. On the contrary, less appreciable changes were noted in untreated control samples (Fig. 3).

### 3.3 Role of cell proliferation in wound healing activity

To analyze the potential contribution of cell proliferation to AMP-induced migration, we initially tested if the well-known cell proliferation inhibitor mitomycin C at 30 μM was sufficient to suppress hDFs proliferation. The proliferation was assessed by Ki-67 immunofluorescence studies. The Ki-67 protein is a popular cellular marker for cell growth and proliferation, being present during all active phases of the cell cycle (G1, S, G2, and M), but absent in quiescent cells (G0) [39]. Fig. S2 shows that treatment of hDFs with 30 μM of mitomycin C for 2 hours significantly reduced the percentage of Ki-67 positive cells with respect to the untreated control samples or to cells treated with the peptides alone.

**Fig. 4:**
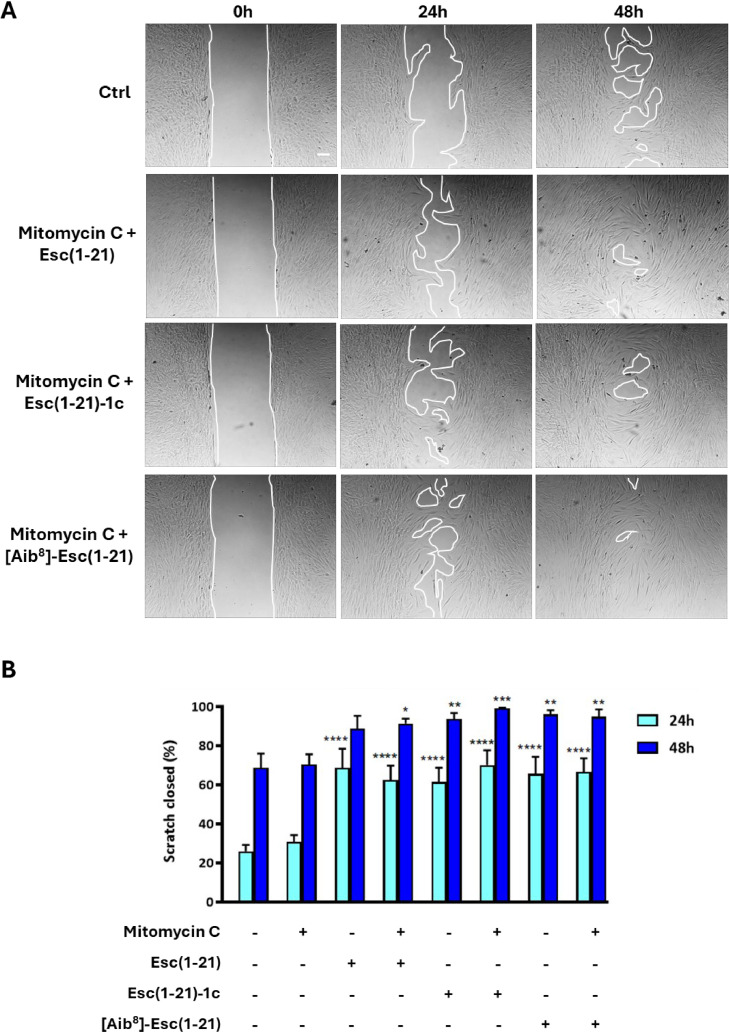
Effect of mitomycin C treatment on peptides-mediated cell migration in hDFs. (A) Representative images of modified scratch assay showing the effect of 10 µM Esc(1-21), Esc(1-21)-1c or [Aib^8^]-Esc(1-21) on hDFs pre-treated with 30 μM mitomycin C for 2 hours (to inhibit cell proliferation). Cells incubated in culture medium alone served as control (Ctrl). Bar: 100 µm. (B) Scratch area closure (%) in fibroblast monolayers pretreated with 30 μM mitomycin C for 2 hours prior to the addition of each peptide at 10 μM. Additional samples included cells treated with peptides or inhibitor alone, and untreated Ctrl cells. The data are the mean from four independent experiments ± SEM. The levels of statistical significance between Ctrl and treated samples at the two time points are indicated as follows: *p <0.05; **, *p*<0.01; ***p <0.001, ****p <0.0001. Statistical analysis revealed no significant differences between peptide-treated samples and samples pre-incubated with mitomycin prior to treatment with the same peptide at either time point.

Subsequently, we explored whether fibroblasts proliferation played a role in the wound closure observed upon exposure to the peptides. To this end, the wound healing assay was performed after pre-treating cells with mitomycin C. As reported in Fig. 4, no statistically significant differences were noted in scratch closure percentages between samples pre-treated with 30 μM mitomycin C for 2 hours prior to exposure to each peptide at 10 μM (the optimal concentration for migration stimulation) and those treated with the peptides alone, at both 24 and 48 hours.

### 3.4 Mechanism of peptide-induced cell migration

**Fig. 5:**
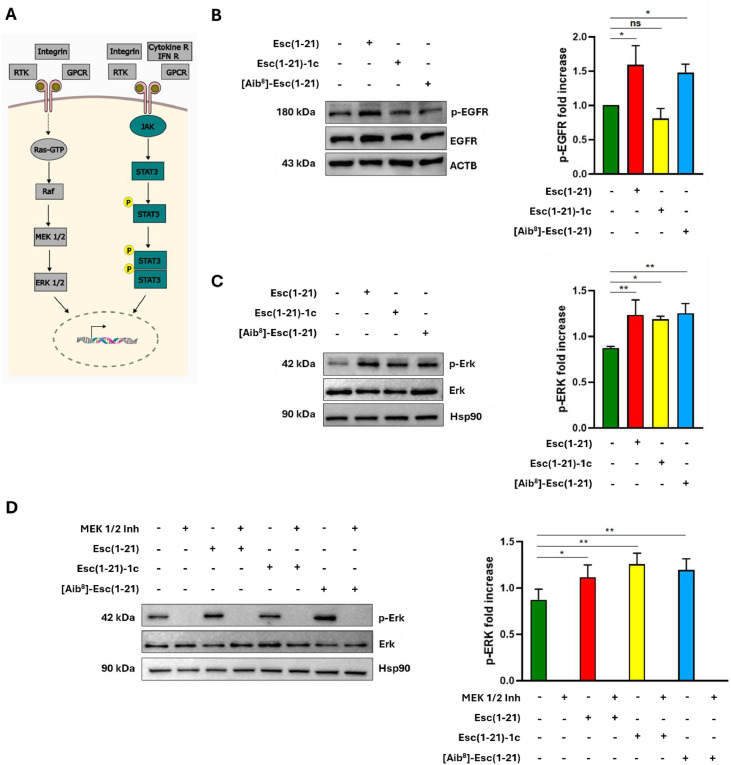
Effect of peptides’ treatment on the activation of the ERK 1/2 pathway. (A) Scheme of the MAPK/ERK and JAK/STAT3 signal transduction pathways. (B) Left: representative Western Blot analysis showing the expression levels of phospho-EGFR (p-EGFR) and total EGFR in hDFs treated for 10 min with 10 µM Esc(1-21), Esc(1-21)-1c or [Aib^8^]-Esc(1-21). Actin B was used as loading control. Right: graph showing densitometric quantification of bands. (C) Left: representative Western Blot analysis showing the expression levels of phospho-Erk 1/2 (p-Erk) and total Erk 1/2 in hDFs treated for 10 min with 10 µM Esc(1-21), Esc(1-21)-1c or [Aib^8^]-Esc(1-21). Hsp90 was used as loading control. Right: graph showing densitometric quantification of bands. (D) Left: representative Western Blot analysis showing the expression levels of phospho-Erk 1/2 (p-Erk) and total Erk 1/2 in presence or absence of MEK 1/2 inhibitor III in hDFs treated for 10 min with 10 µM Esc(1-21), Esc(1-21)-1c or [Aib^8^]-Esc(1-21). Hsp90 was used as loading control. Right: graph showing densitometric quantification of bands. Data are shown as mean fold increase ± standard deviation (SD) from three independent experiments and the levels of statistical significance between Ctrl and treated samples are indicated as follows: *p <0.05 and ** *p* <0.01. ns, not significant.

It is well known that migration of several cell types, including skin cells such as keratinocytes and dermal fibroblasts, is predominantly regulated by the activation of receptor tyrosine kinases (RTKs, such as EGFR, FGFR, and PDGFR), integrins, and G protein–coupled receptors, which converge on the intracellular MAPK/ERK cascade or can activate JAK/STAT3 pathway [[40], [41], [42], [43], [44], [45]] (Fig. 5A).

In our previous work, it was demonstrated that Esc(1-21) markedly promotes the migration of human immortalized keratinocytes through EGFR activation [28]. Therefore, to assess whether a similar mechanism underlined migration of dermal fibroblasts following treatment with the selected peptides, the phosphorylation level of EGFR was evaluated by western blot analyses as a readout of receptor activation after peptide treatment. As indicated in Fig. 5B, both Esc(1-21) and [Aib^8^]-Esc(1-21) at 10 μM significantly enhanced EGFR phosphorylation after 10 min, while no difference was observed for the diastereomer Esc(1-21)-1c.

Subsequently, to determine the involvement of MAPK/ERK signaling pathway, and specifically of the extracellular signal-regulated kinase (ERK) 1/2 [10,11], the level of ERK1/2 phosphorylation was monitored. According to the results of western blot analysis, all three peptides increased ERK1/2 phosphorylation after 10 min (Fig. 5C). Moreover, pre-treatment with MEK1/2 inhibitor III, which targets upstream kinases of ERK1/2, completely abolished both basal and peptide-induced ERK1/2 phosphorylation (Fig. 5D).

Furthermore, to verify whether additional signaling pathways downstream RTKs were implicated in the peptide-mediated migration process (Fig. 5A), the activation of the signal transducer and activator of transcription 3 (STAT3) [28,46,47] was investigated by analyzing its phosphorylation level. As reported in Fig. S3, all the three AMPs did not significantly increase STAT3 phosphorylation at the same time point.

**Fig. 6:**
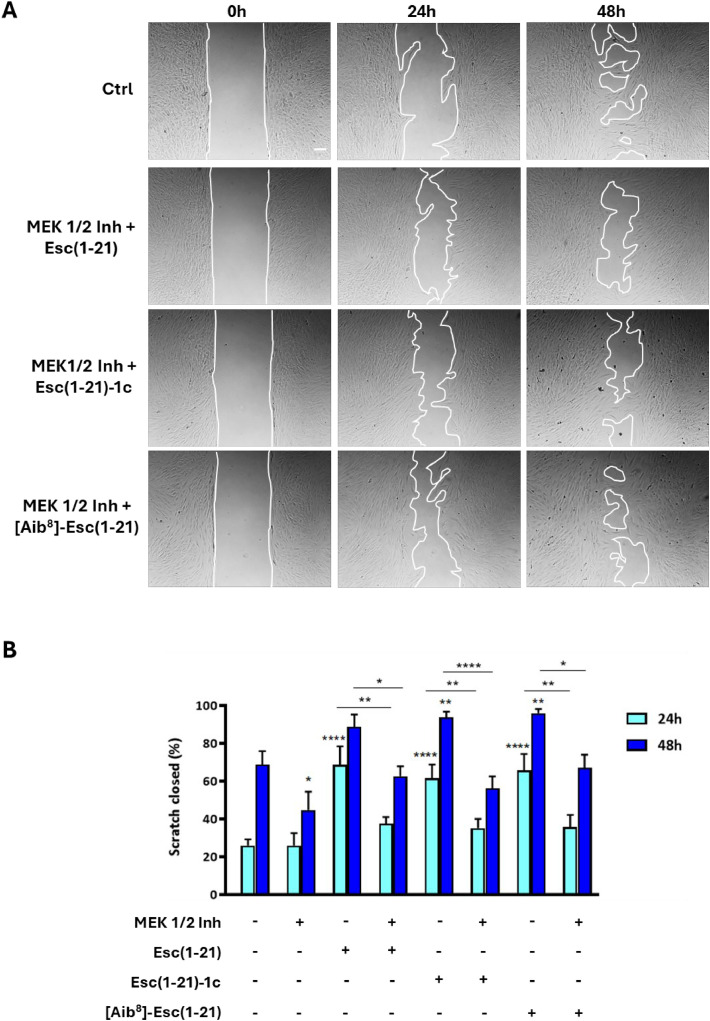
Impact of MEK1/2 pathway inhibition on peptides-dependent cell migration in hDFs. (A) Representative images of modified scratch assay showing the effect of 10 µM of each peptide on fibroblasts migration, following 1 h pre-treatment with MEK 1/2 inhibitor III. Cells incubated in culture medium alone served as control (Ctrl). Bar: 100 µm. (B) Scratch area closure (%) in a monolayer of fibroblasts pretreated with 1 μM MEK1/2 Inhibitor III for 1 hour and subsequently exposed to each peptide at 10 μM in the presence of the inhibitor. Additional samples included cells treated with peptides or inhibitor alone, and untreated Ctrl cells. The data are the mean from four independent experiments ± SEM. The levels of statistical significance between Ctrl and treated samples at the two time points are indicated as follows: *p <0.05; **p <0.01; ****p <0.0001. Significance between peptide-treated samples and samples incubated with MEK inhibitor in the presence of the same peptide at both time points is shown on the connecting lines (*p <0.05; **p <0.01, ****p <0.0001).

**Fig. 7:**
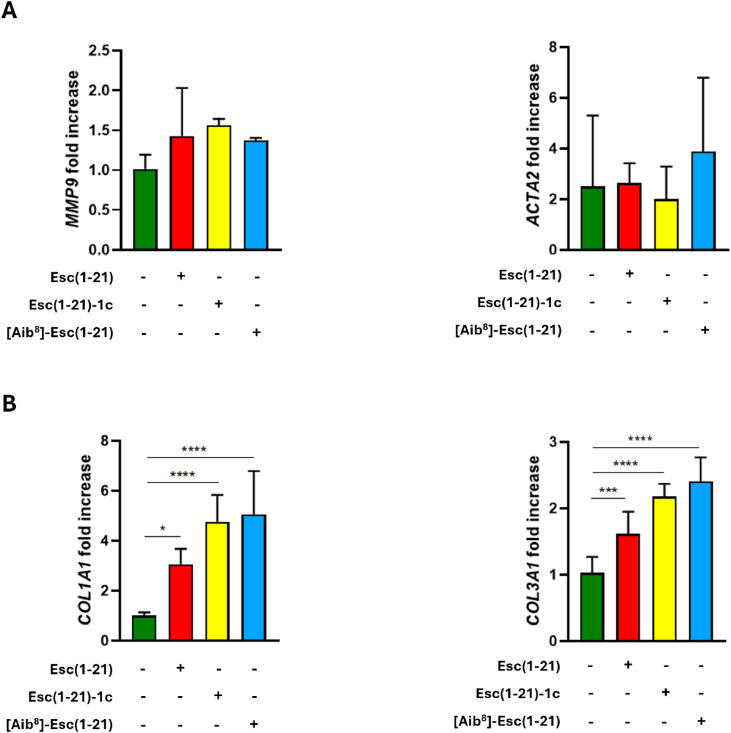
Effect of peptides on *MMP9, ACTA2, COL1A1* and *COL3A1* gene expression in hDFs. Effect of peptides’ treatment on *MMP9* and *ACTA2* (A), or *COL1A1* and *COL3A1* (B) mRNA expression in hDFs treated as above for 12 hours. Cells were lysed and the extracted RNA was analyzed by real-time PCR. Results are expressed as fold increase of mRNA expression with respect to the untreated Ctrl and indicated as the mean value ± SD of three independent experiments. Significance levels are indicated as follows: *p* <0.05 (*), <0.001 (***) and <0.0001 (****).

Subsequently, to assess whether the peptide-induced migration of fibroblasts depended on ERK1/2 signaling, cells were pretreated with 1 μM MEK1/2 inhibitor III (which blocks ERK1/2 phosphorylation), prior to the addition of each peptide in combination with the inhibitor. The peptides-induced increase in fibroblast migration was significantly reverted by MEK 1/2 inhibition at both time points, compared to the untreated control cells (Fig. 6). To further explore the wound healing potential of the three peptides, their effect on the transcriptional expression of key molecules employed in fibroblast-mediated tissue repair was then examined. The gene expression of matrix metalloprotease-9 (MMP9), an enzyme implied in extracellular matrix remodelling and collagen degradation, was assessed. Treatment of hDFs with the peptides did not affect MMP9 expression at either the gene level (Fig. 7A) or the protein level (Fig. S4). Fibroblasts activation, commonly associated with their role in tissue repair, was evaluated via expression of the α-smooth muscle actin (α-SMA) marker. Likewise, no significant changes in α-SMA expression were observed at the transcript level (*ACTA2*) (Fig. 7A) or protein level (Fig. S4) following peptide treatment.

In addition, the expression of collagen types most prominently involved in fibroblast-driven wound healing was also determined. A significant increase of type I and III collagen mRNA expression (*COL1A1* and *COL3A1*) was observed in cells treated with the peptides compared to untreated control cells (Fig. 7B). Moreover, our data demonstrated that [Aib⁸]-Esc(1-21) significantly increased *COL1A1* expression compared to Esc(1-21) (*p* < 0.05), while *COL3A1* expression was significantly enhanced by both Esc(1-21)-1c (*p* < 0.01) and [Aib⁸]-Esc(1-21) (*p* < 0.0001) relative to Esc(1-21).

## 4 Discussion

Skin wound healing is a complex, multi-step process involving the interplay of different cell types, including keratinocytes, endothelial cells, pericytes, immune cells and fibroblasts. Fibroblasts, in particular, are critical players in tissue repair, as they rebuild the extracellular matrix (ECM), drive wound contraction, and coordinate repair. Despite their central role, relatively little is known about how AMPs directly affect fibroblast functions during wound healing. Most studies have focused instead on keratinocytes or endothelial cells [28,[48], [49], [50], [51], [52], [53]]. Addressing this gap, the present study aimed to investigate the functional contribution of the frog-skin derived AMPs Esc(1-21), Esc(1-21)-1c and [Aib^8^]-Esc(1-21) to hDFs activation during skin repair.

Both cell migration and proliferation are essential processes to initiate and complete wound closure [54,55]. For the first time, we showed that both Esc peptides and the isoform [Aib^8^]-Esc(1-21) significantly enhance hDFs migration in a pseudo-wound healing assay, at both 24 or 48 hours with clear efficacy at 10 µM and partial effects even at 4 µM (Fig. S1). Crucially, this activity is not accompanied by cytotoxicity: none of the peptides compromised cell viability up to 16 µM (Fig. 1), and Esc(1-21)-1c displayed no toxicity even at 128 µM, consistent with its distinct conformation and reduced affinity for mammalian membranes [23]. Furthermore, wound closure was not impaired by pre-treatment with the cytostatic agent mitomycin C, strongly suggesting that Esc peptide-mediated repair is primarily driven by cell migration rather than proliferation (Fig. 4A-B).

Overall, these data differ from many previous studies where AMP-mediated effects on cells proliferation and migration were analyzed separately or not mechanistically dissected. For instance, cathelicidin-DM, Clavanins, and Mastoparan-MO have been reported to stimulate fibroblast proliferation and migration, but without clarifying the relative contribution of these processes [7,56]. Similarly, LL-37 has been shown to stimulate keratinocyte and endothelial cell repair [47,[57], [58], [59], [60]]. However, in many of these studies, proliferation was assessed with general assays (e.g., MTT), rather than in combined experimental settings such as wound healing assays performed in the presence of proliferation inhibitors, as in our case. Thus, the interplay between proliferation and migration remains unresolved [47,[57], [58], [59], [60]]. Here we provide evidence that Esc peptides and [Aib^8^]-Esc(1-21) promote fibroblast migration without affecting cell’s proliferation, clarifying an important aspect in understanding AMP activity during fibroblast-mediated wound healing. Notably, the possibility that the stimulation of fibroblast migration might arise from a non-specific cationic coating of the culture plastic surface, similar to poly-L-lysine, appears unlikely. Previous structure–activity relationship studies on esculentin-1-derived peptides have shown that their biological activity cannot be explained solely by their cationicity. Only the all-L form of Esc(1-21), but not its all-D enantiomer (despite identical length, amino acid composition, and net charge) was able to stimulate human keratinocyte migration in vitro, supporting a stereospecific mechanism that is inconsistent with a simple polycationic surface-coating effect [28]. Moreover, the unrelated cationic peptide, colistin, failed to induce epithelial cell migration under comparable experimental conditions as Esc(1-21) [24].

In addition to the functional effect, we explored the molecular basis of Esc peptide-induced fibroblast activation. We and other authors reported that migration of epithelial cells stimulated by frog-skin derived AMPs during re-epithelization relies on EGFR [28,50,61]. Furthermore, Esc(1-21) was reported to promote keratinocyte migration through activation of STAT3 protein [28]. However, EGFR activation can trigger distinct intracellular transduction pathways [62] (Fig. 5A), especially those employing MAP kinases, that have been shown to play an important role in regulating keratinocytes and fibroblasts’ migration [10,11].

In this work, we discovered that treatment of hDFs with Esc peptides or the [Aib^8^]-Esc(1-21) rapidly led to a robust phosphorylation of ERK1/2 (Fig. 5C), which was fully prevented by a MEK inhibitor (Fig. 5D), demonstrating that MAPK/ERK signaling is required for Esc-mediated migration of hDFs (Fig. 6A-B). Our data have also indicated that a strong phosphorylation of EGFR is not strictly required for the observed pro-repair activity of fibroblasts. Indeed, in contrast with what observed for Esc(1-21) and [Aib^8^]-Esc(1-21) peptides, Esc(1-21)-1c did not appear to significantly increase EGFR phosphorylation compared to the untreated samples (Fig. 5B). It should be considered that, according to what is reported in the literature, functional involvement of EGFR does not necessarily imply a strong increase in its phosphorylation detectable by western blot at a single time point [63]. In addition, EGFR activation may be transient but still sufficient to sustain downstream signaling. Furthermore, the pronounced EGFR phosphorylation observed with Esc(1-21) and [Aib⁸]-Esc(1-21) may partially reflect their mildly cytotoxic profiles, as cellular stress and ROS can modulate protein tyrosine phosphatases, thereby increasing the magnitude of EGFR phosphorylation [64]. Given that all peptides converged on the activation of MAPK via ERK phosphorylation, it is possible that Esc(1-21)-1c activates ERK through transient EGFR-dependent mechanisms and/or through parallel intracellular pathways triggered by other RTKs (e.g. FGFR) or by recruitment of specific protein adaptors before the activation of ERK. The implication of RTKs other than EGFR upon AMPs treatment was indeed described in fibroblasts, as in the case of human β-Defensin 3 promoting fibroblast migration through Fibroblast Growth Factor 1 (FGF1) [65]. Alternatively, different types of receptors, including integrins or G-protein coupled receptors (GPCR) may participate in Esc(1-21)-1c mediated ERK activation. Moreover, the absence of significant STAT3 phosphorylation (Fig. S3) means that, although alternative signaling branches involving this protein may be engaged, they are not essential to boost fibroblast migration. Instead, ERK activation appears sufficient to sustain this biological response, regardless of the contribution of other intracellular pathways. The observed differences among the selected peptides are likely related to their structural features. The presence of D-amino acids in Esc(1-21)-1c may influence peptide–protein interactions through stereospecific recognition, potentially altering binding to different receptors or intracellular adaptor proteins. The differential effects may therefore arise from the combined influence of multiple factors, encompassing variations in membrane interactions, cytotoxicity, signaling kinetics, and activation of convergent pathways upstream of ERK. To note, although MAPK-mediated signaling pathways are commonly associated with inflammatory responses [66,67], the activation observed in fibroblasts is not expected to translate into pathological inflammation. Previous studies have shown that Esc peptides inhibit TNF-α and IL-6 secretion in lipopolysaccharide-stimulated murine macrophages [29,68] and do not induce detectable inflammatory alterations in mouse lungs following intratracheal administration at therapeutically relevant doses [69]. These findings support a context-dependent, signalling response that promotes tissue repair rather than inflammation.

Remarkably, fibroblasts are pivotal to wound closure but also to subsequent ECM deposition and remodeling. In this respect, our results revealed that Esc peptides and [Aib^8^]-Esc(1-21) selectively upregulate early ECM genes, such as Collagen I and III, without increasing ACTA2 (α-SMA) or MMP9 expression (Fig. 7A-B) (Fig. S4), nor eliciting fibroblast differentiation into contractile myofibroblasts [38]. The results presented in Fig. 7B highlight that differences among peptides are likely related to variations in proteolytic stability among them. Both Aib incorporation and L-to-D amino acid substitution are known to enhance resistance to proteolytic degradation, thereby prolonging peptide lifetime and bioavailability [29,31], which may contribute to the greater upregulation of collagen gene expression by Esc(1-21)-1c and [Aib⁸]-Esc(1-21). However, the lack of a significant effect of Esc(1-21)-1c on COL1A1 compared to Esc(1-21) suggests that increased stability alone does not fully account for the observed differences. This indicates that in addition to proteolytic stability, specific structural features of the analogues may modulate their interactions with cellular targets and the activation of downstream signaling pathways regulating collagen gene expression.

Altogether, these results point out that the selected peptides activate fibroblasts toward an early wound healing phenotype, facilitating matrix deposition without promoting late-stage contractile or ECM degradation programs [70] (Fig. 7A). These data align with previous findings on other AMPs, such as Clavanins, Mastoparan-MO or LL-37, which were shown to induce fibroblast migration and to regulate the expression of specific genes, such as collagen, in a dose and context-dependent manner [56,71]. Notably, in pathological settings characterized by impaired wound healing, the same AMP may influence the process in a markedly different manner. For instance, skin keloids are fibroproliferative dermal disorders characterized by excessive deposition of collagen that fails to regress over-time [72]. LL-37 expression was reduced in the skin of keloid patients and treatment of keloid dermal fibroblasts with LL-37 decreased *in vitro* the transcription of collagen genes as well as the deposition of collagen, thus acting as anti-fibrotic agent [73].

Altogether, our findings identified dermal fibroblasts as novel direct targets of Esc peptides, complementing previous knowledge that focused mainly on keratinocytes and endothelial cells [28,[48], [49], [50], [51], [52], [53]]. By uncovering a non-toxic, ERK-dependent enhancement of fibroblast migration and early ECM gene expression, this work provides mechanistic evidence that Esc peptides isoforms can positively modulate key fibroblast functions required for tissue repair. Importantly, these results suggest that Esc peptide isoforms can have therapeutic potential in conditions where fibroblast activation and wound healing are impaired.

## 5 Conclusion

In conclusion, this study demonstrates for the first time that frog-skin derived Esc peptide isoforms (i) directly stimulate fibroblast migration primarily through MAPK/ERK activation mediated by EGFR, although the contribution of other intracellular pathways cannot be excluded at this stage; (ii) enhance expression of early ECM genes, without inducing proliferation, cytotoxicity or fibroblast-to-myofibroblast transition. These findings expand our understanding of AMP biology in wound healing, underscoring fibroblasts as critical cellular targets, and open new perspectives for the therapeutic use of these peptides in regenerative medicine.

## Funding

This work was supported by European Union’s NextGeneration EU‐MUR PNRR Extended Partnership initiative on Emerging Infectious Diseases (Grant Project n. PE00000007, INF‐ACT node 3 to M.L.M.) and partially supported from Sapienza University (Grant N. RG124190CBD34B9D).

## Ethics declarations

The use of clinical samples of dermal tissue for fibroblast isolation complied with the Declaration of Helsinki 1975, revised in 2008. Written consent was obtained from all subjects.

## Ethics approval and consent to participate

Institutional Review Board Statement: This study was approved by the Institutional Ethics Committee of Sant’Andrea Hospital (CE/564/11), 10 October 2011.

## CRediT authorship contribution statement

**Floriana Cappiello:** Writing – original draft, Methodology, Investigation, Formal analysis. **Eleonora Grisard:** Writing – review & editing, Writing – original draft, Visualization. **Alice Traversa:** Investigation. **Danilo Ranieri:** Writing – original draft, Investigation, Formal analysis, Conceptualization. **Maria Luisa Mangoni:** Writing – review & editing, Writing – original draft, Supervision, Project administration, Funding acquisition, Conceptualization.

## Declaration of competing interest

The authors declare that they have no known competing financial interests or personal relationships that could have appeared to influence the work reported in this paper.

## Data Availability

All data are available upon request to the authors.

## Appendix Supplementary materials

Appendix A. Supplementary data Figure S1. Impact of peptides’ treatment on the migration of hDFs; Figure S2. Evaluation of mitomycin C effect on cell proliferation in hDFs; Figure S3. Effect of peptides’ treatment on the activation of the STAT3 pathway; Figure S4. Effect of peptides on MMP9 and α-SMA protein expression in hDFs. Image, application 1
