# Supplementary material for: Bioactive derivatives of the antimicrobial peptide esculentin-1a promote human dermal fibroblast migration and activate genes involved in early wound healing
Source: BBA Adv. 2026 Mar 28;9:100186. doi: 10.1016/j.bbadva.2026.100186 (PMC13087719; doi:10.1016/j.bbadva.2026.100186)
Supplement: Supplementary file 1 — Appendix A. Supplementary data Figure S1. Impact of peptides’ treatment on the migration of hDFs; Figure S2. Evaluation of mitomycin C effect on cell proliferation in hDFs; Figure S3. Effect of peptides’ treatment on the activation of the STAT3 pathway; Figure S4. Effect of peptides on MMP9 and α-SMA protein expression in hDFs. [file mmc1.docx]

**Appendix A. Supplementary data**

**Bioactive derivatives of the antimicrobial peptide esculentin-1a promote human dermal fibroblast migration and activate genes involved in early wound healing**

Floriana Cappiello*, Eleonora Grisard, Alice Traversa, Danilo Ranieri^#^, Maria Luisa Mangoni^1#^*

* corresponding author

# contributed equally to this work

^a^Laboratory Affiliated to Pasteur Italia-Fondazione Cenci Bolognetti, Department of Biochemical Sciences, Sapienza University of Rome, 00185 Rome, Italy;

^b^Department of Life Science, Health, and Health Professions, Link Campus University, 00165 Rome, Italy.

* Corresponding author at: Department of Biochemical Sciences, Sapienza University of Rome, 00185 Rome, Italy. *E-mail addresses*: marialuisa.mangoni@uniroma1.it, Tel.: +39-0649910838 (M.L. Mangoni); floriana.cappiello@iss.it (F. Cappiello)

**The supplementary information includes:**

**Fig S1-S4**


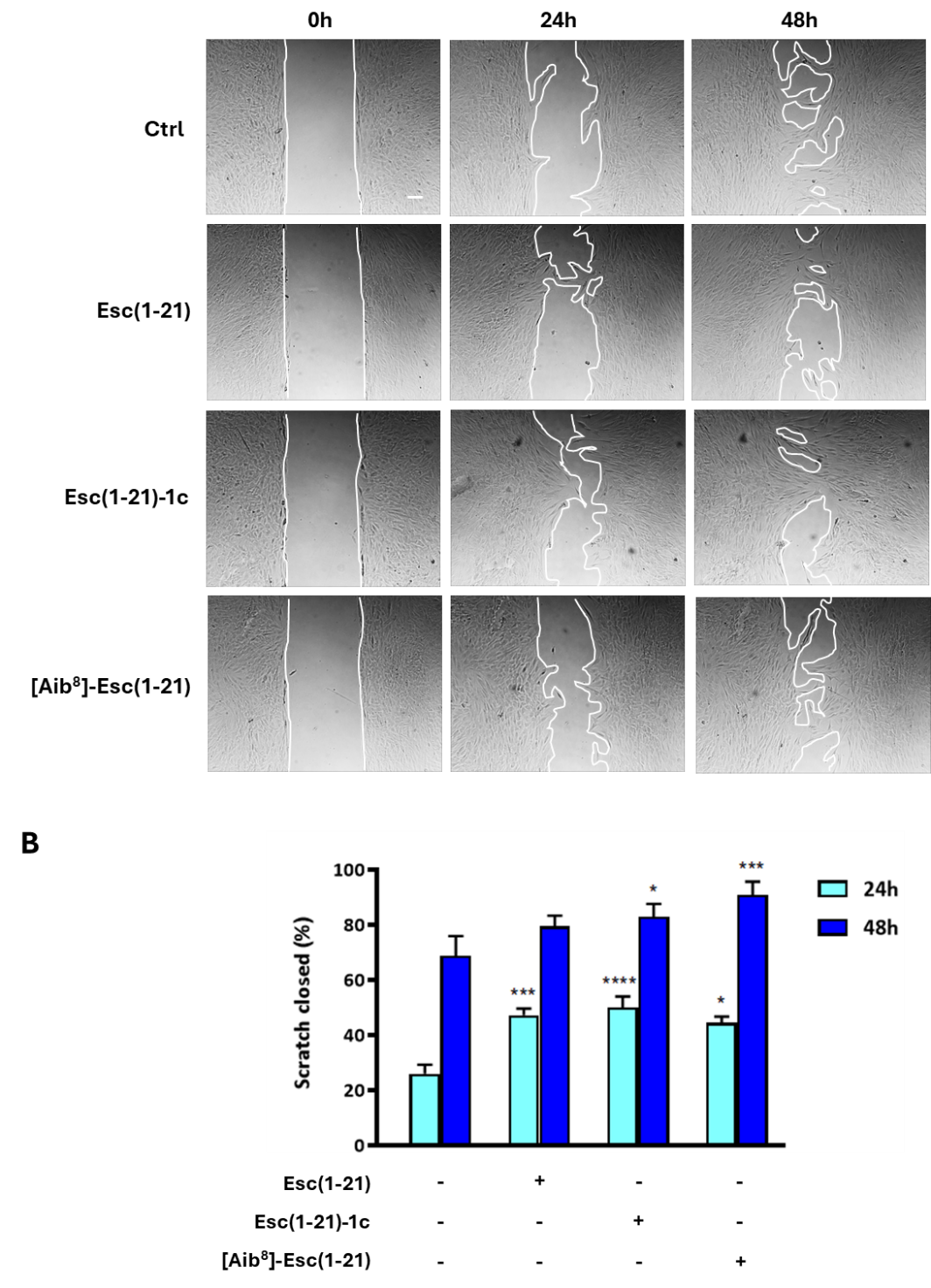


**Fig. S1 Impact of peptides’ treatment on the migration of hDFs. (A)** Representative images from the modified scratch assay showing the effects of treatment of fibroblast monolayers with 4 µM Esc(1-21), Esc(1-21)-1c or [Aib^8^]-Esc(1-21). The peptides promoted a significant reduction in the residual open area and induced a typical migratory phenotype, after 24 and 48 hours of treatment. Peptide-untreated cells were used as a control (Ctrl). Bar: 100 µm. **(B)** Scratch area closure (%) in fibroblast monolayers, measured after 24- and 48-hours treatment with 4 µM peptides to evaluate cell migration. The data are the means from four independent experiments ± SEM. The levels of statistical significance between Ctrl and treated samples are indicated as follows: p values <0.05 (*), <0.001 (***), and <0.0001 (****).


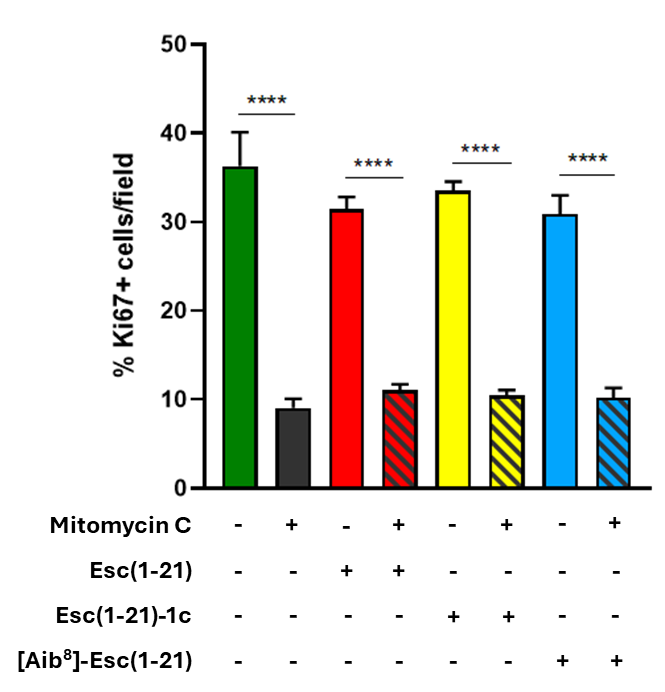


**Fig. S2** Evaluation of mitomycin C effect on cell proliferation in hDFs. Quantitative analysis of cell proliferation was performed by counting Ki-67 positive cells following treatment with 30 µM mitomycin C alone and/or in combination with 10 µM Esc(1-21), Esc(1-21)-1c or [Aib^8^]-Esc(1-21). Data are shown as mean percentages ± SEM from three independent experiments. Statistical significance between groups is indicated as follows: p values <0.0001 (****).


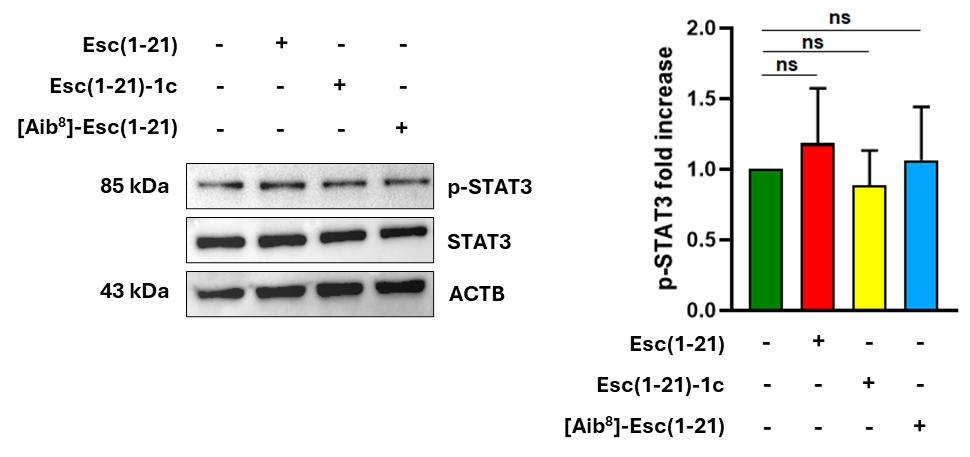


**Fig. S3 Effect of peptides’ treatment on the activation of the STAT3 pathway.** Fibroblasts were treated for 10 min with each peptide at 10 μM. Western blot analysis was performed using an antibody specific for the phosphorylated form of STAT3. Equal protein loading was verified using β-actin antibody as loading control. Densitometric quantification and statistical analysis were performed as reported in Materials and Methods. Data are shown as mean fold increase ± standard deviation (SD) from three independent experiments and the levels of statistical significance are indicated. ns, not significant.


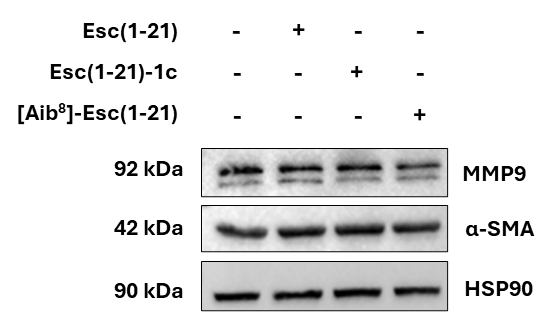


**Fig. S4 Effect of peptides on MMP9 and** α**-SMA protein expression in hDFs.** Primary fibroblasts cultures were treated for 24 hours with each peptide at 10 μM. Western blot analysis was performed using an antibody specific for α-SMA or MMP9. Equal protein loading was verified using HSP90 antibodies as loading control.
